# Post-Transcriptional Inflammatory Response to Intracellular Bacterial c-di-AMP

**DOI:** 10.3389/fimmu.2019.03050

**Published:** 2020-01-17

**Authors:** Linah Mahmoud, Alaa S. Abdulkarim, Shaima Kutbi, Walid Moghrabi, Sulaiman Altwijri, Khalid S. A. Khabar, Edward G. Hitti

**Affiliations:** Molecular BioMedicine Program, King Faisal Specialist Hospital & Research Centre, Riyadh, Saudi Arabia

**Keywords:** c-di-AMP, p38 MAPK, tristetraprolin/zink finger protein 36, AU-rich element, post-transcriptional regulation

## Abstract

Cyclic-di-AMP (c-di-AMP) is a bacterial second messenger that is produced by intracellular bacterial pathogens in mammalian host macrophages. Previous reports have shown that c-di-AMP is recognized by intracellular pattern recognition receptors of the innate immune system and stimulate type I interferon response. Here we report that the response to c-di-AMP includes a post-transcriptional component that is involved in the induction of additional inflammatory cytokines including IL-6, CXCL2, CCL3, and CCL4. Their mRNAs contain AU-rich elements (AREs) in their 3′ UTR that promote decay and repress translation. We show that c-di-AMP leads to the phosphorylation of p38 MAPK as well as the induction of the ARE-binding protein TTP, both of which are components of a signaling pathway that modulate the expression of ARE-containing mRNAs at the post-transcriptional level. Pharmacological inhibition of p38 reduces the c-di-AMP-dependent release of induced cytokines, while TTP knockdown increases their release and mRNA stability. C-di-AMP can specifically increase the expression of a nano-Luciferase reporter that contains AREs. We propose a non-canonical intracellular mode of activation of the p38 MAPK pathway with the subsequent enhancement in the expression of inflammatory cytokines. C-di-AMP is widely distributed in bacteria, including infectious intracellular pathogens; hence, understanding of its post-transcriptional gene regulatory effect on the host response may provide novel approaches for therapy.

## Introduction

Cyclic dimeric AMP (c-di-AMP) or bis-(3′,5′)-cyclic dimeric adenosine monophosphate is a bacterial second messenger that is involved in a number of bacterial metabolic events such as DNA damage-dependent cell cycle control, establishment of infection, cell size control, osmotic homeostasis, and regulation of metabolic enzyme function (1–8). C-di-AMP produced by intracellular bacterial pathogens can be recognized by cytoplasmic pattern recognition receptors of the mammalian innate immune system and induces type I interferon response in macrophages (9, 10).

The mRNAs of interferons, like those of many other cytokines, contain AU-rich elements (AREs) in their 3′untranslated regions (3′UTR). AREs reduce the stability and translation of cis-mRNAs; a process that maintains low or no expression of the inflammatory cytokines when inflammatory response is not needed, but they rapidly and transiently respond to inflammatory stimuli and drive expression at the post-transcriptional level (11, 12). The main cellular signaling event that targets AREs to de-repress mRNA expression is the p38 mitogen-activated-protein-kinase (MAPK) signaling pathway that responds to inflammatory stimuli and stress (13–15).

The canonical activation of the post-transcriptional signaling pathway of p38 MAPK in inflammation starts by the activation of cell surface receptors like cytokine and Toll-like receptors that activate TNF-receptor-associated factors (16, 17). This is followed by the phosphorylation-dependent activation of a MAP Kinase Kinase Kinase (MAP3K) such as TAK1 (MAP3K7). MAP3Ks in turn phosphorylate MAP2Ks such as MKK6 and MKK3, leading to the phosphorylation and activation of the central element of the pathway, MAPK p38 (18–20). In turn, p38 can phosphorylate and activate a number of downstream kinases such as MK2 and MK3 (14, 21, 22). The link between MK2 and target mRNAs is the AU-rich elements binding protein tristetraprolin (TTP; also called ZFP36). Not only the activity of p38 and its downstream kinase MK2 is required for the expression but also the inhibition of TTP activity by direct phosphorylation. For instance, the treatment of macrophages by lipopolysaccharide (LPS) leads to a strong induction of TTP; however, this induction can be reduced by the p38 inhibitor SB203580 or in an MK2 knockout background. The low levels of TTP in a p38 inhibition background is believed to be more active than the highly expressed and phosphorylated TTP (23–27).

Alternative models of activation of p38 have been reported, including intracellular activation. Molecules that can be recognized by cytoplasmic pattern recognition receptors such as RIG-I or NOD1 were shown to activate p38 (28–32).

Here we report that intracellular c-di-AMP can induce the expression of a subset of inflammatory ARE-containing mRNAs at the transcriptional and post-transcriptional levels.

## Materials and Methods

### RAW 264.7 and Bone-Marrow-Derived Mouse Macrophage Preparation

RAW 264.7 cell lines were purchased from American Type Culture Collection (ATCC; Rockville, MD) and grown in DMEM medium supplemented with 10% heat-inactivated fetal calf serum (FCS) and antibiotics (Invitrogen, Carlsbad, CA). Bone-marrow-derived macrophages (BMDM) were prepared as described previously (25). Briefly, bone marrow from femurs of WT mice were flushed with DMEM medium supplemented with FCS. An amount of 10 ng of M-CSF/ml was added to induce macrophage differentiation; material from one mouse was cultured in two 10-cm plates for 9–10 days.

### Intracellular Delivery of c-di-AMP and SB203580 Treatment

Macrophages were cultured in six-well plates to ~80% confluence, medium was removed, and 2.5 μM of c-di-AMP was added in 0.5-ml permeabilization solution (PS) (50 mM HEPES pH 7.0, 100 mM KCl, 3 mM MgCl2, 0.1 mM DTT, 85 mM sucrose, 0.2% BSA, 1 mM ATP, 0.1 mM GTP, and 10 μg/mL digitonin). PS/c-di-AMP mix was left on cells for 30 min and then removed, and growth medium was added. Typically, the p38 inhibitor SB203580 (Promega, catalog #V1161) was added at a final concentration of 5 μM with the c-di-AMP/PS mix. Dimethyl sulfoxide (DMSO, Sigma, catalog #D2650) was used as vehicle control. After the removal of c-di-AMP/PS, 5 μM SB203580 or equivalent DMSO was again added with medium to cells.

### RNA Preparation, Real-Time PCR, and Actinomycin-D Chase

Total RNA was extracted with TRI reagent (Sigma). Reverse transcription was performed using Superscript II and Oligo dT primer (Invitrogen) as described previously (27). Real-time PCR TaqMan primer sets including the VIC-labeled β-actin as internal control as well as FAM-labeled primer sets for mouse IFNβ, IL6, TNF, COX-2, CCL3, CCL4, CXCL10, and CXCL2 were ordered from Applied Biosystems. Real-time PCR was performed using the CFX96 cycler (BioRad). For mRNA half-life determination, cells were treated with Actinomycin-D (10 μg/ml) to shut off transcription. For half-life experiments with the p38 inhibition, the inhibitor SB203580 (or corresponding DMSO vehicle) was added at a concentration of 5 μM with the c-di-AMP/PS mix. The c-di-AMP/PS mix was removed, and 5 μM SB203580 or DMSO was again added with new growth medium to cells. After 4 h of induction, Actinomycin-D was added to shut off transcription. Decay curves were plotted using GraphPad Prism software. Estimated half-life is the intersection point between the decay curve and the 0.5 fraction remaining.

### ELISA and Multiplex ELISA

Single-assay ELISA kits were purchased from Thermo Scientific, and experiments were performed according to the manufacturer's protocol. For multiplex ELISA, MILLIPLEX MAP Mouse Cytokine/Chemokine Magnetic Bead Panel (MCYTOMAG-70K) was purchased from Millipore, and experiments were performed according to the manufacturer's protocol using Luminex Bio-Plex 200 system.

### TTP Knockdown

siTTP (as four pooled siRNAs) and control non-targeting siRNA (SCR) were ordered from Dharmacon (Cat. Nb. A- E-041045-00-0005; Cat. Nb. D-001210-01-20) and were previously validated (27). Typically, RAW264.7 cells were transfected with 50 nM siRNA using Lipofectamine LTX (Thermofisher) for 48 h. The efficiency of the knockdown (KD) was assessed by western blotting.

### Western Blotting and Calf Intestinal Phosphatase Treatment

For total lysates, cells were directly lysed in 2 × SDS sample buffer (Invitrogen). Lysates were sonicated to shear DNA, and equivalent lysate levels were loaded onto SDS PAGE gel, blotted to nitrocellulose membrane, and probed with antibodies to TTP, phospho p38 (P-P38), phospho-MK2, MK2, and β-actin. Affinity-purified TTP rabbit polyclonal antibody was custom-made with Genescript against the C-terminal end of TTP PRRLPIFNRISVSE and was used previously (27). The specificity of the antibody was tested in western blots from transfected and/or LPS-induced cells and with pre-immune serum. P38 antibody was purchased from Santa Cruz Biotechnology; anti-phospho-p38 (Thr180/Tyr182) was purchased from Millipore/Merck; β-actin, MK2, and P-MK2 (Thr334) antibodies were purchased from Cell Signaling. For calf intestinal phosphatase (CIP) (Promega) treatment, cells were lysed in 1 × CIP buffer supplemented with 0.5% NP40. Typically, 100 μl of lysate was treated with 10 units of CIP for 30 min at 37°C.

### Reporter Assays

RAW264.7 cells were seeded in a 10-cm plate and co-transfected using Lipofectamine LTX with 4 μg nano-Luciferase (nLuc) reporters and 4 μg Firefly (FF) reporter. The 3′UTR of the mouse *CCL3* gene was amplified by PCR and cloned into the *Bam*HI and *Xba*I sites of the 3′UTR region of the nLuc reporter vector that was previously used (27) (forward primer: AGCGGATCCGAGTCTTGGAGGCAGCGAGGA, reverse primer: AGCTCTAGACACTTGTTAAAGGGCATATTTAT). Following overnight incubation, cells were treated with either PS alone, c-di-AMP in PS, or LPS or left untreated and then incubated for 8 h in fresh serum-free medium. Dual nano-Luciferase assays were performed using the kit and protocol of ONE-Glo™ EX Luciferase Assay System from Promega. For reporter assays with siRNA, 50 nM end concentration of siRNA was cotransfected with the DNA mix.

## Results

### c-di-AMP Induces a Subset of ARE-Containing mRNAs

**Figure 1:**
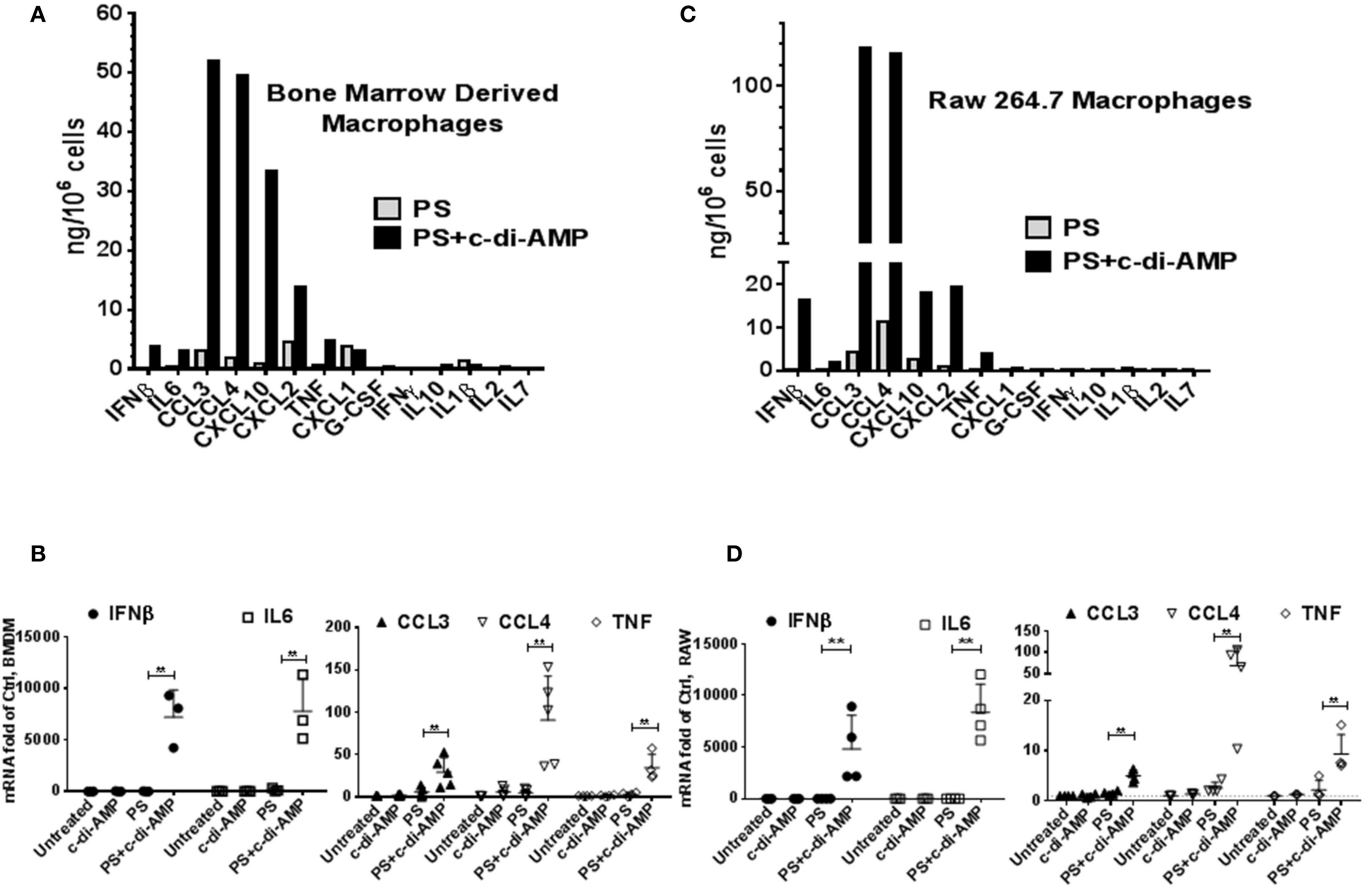
c-di-AMP induces a subset of inflammatory cytokines. **(A)** 5 × 10^5^ mouse BMDM in six-well plates were treated with PS alone or PS+c-di-AMP for 4 h; supernatants were recovered, and simple IFNβ and multiplex ELISAs were performed. **(B)** Total RNA was prepared and quantitative real-time PCR was performed for the indicated mRNAs with additional controls. Cells were left untreated, treated with c-di-AMP alone, PS alone, and PS+c-di-AMP. **(C)** A similar ELISA experiment was performed with RAW264.7 macrophages. **(D)** RT-PCR for the indicated mRNAs from RAW264.7 cells. Individual values of at least three independent experiments are represented as fold of untreated control. Means ± SEMs are shown. *Asterisk* represents *P* <0.05 of paired *t*-tests.

c-di-AMP induces IFNβ in macrophages (2). To investigate whether c-di-AMP is capable of inducing other cytokines, we treated BMDM either with PS alone or with PS and c-di-AMP for 4 h and conducted multiplex ELISA on the supernatant culture medium using multiplex cytokine/chemokine magnetic bead panel that quantifies the release of ~30 cytokines. Since the kit does not contain IFNβ, additional IFNβ ELISA was performed in parallel (Figure 1A). The c-di-AMP molecules specifically induced several other cytokines like IL6, CCL3, CCL4, CXCL10, CXCL2, and TNF, while CXCL1, G-CSF, IFNγ, IL10, IL1β, IL2, and IL7 were not induced. The levels of the remaining cytokines from the panel were below detection limit and include Eotaxin, G-CSF, GM-CSF, IL-1α, IL-2, IL-3, IL-4, IL-5, IL-7, IL-10, IL-12, IL-12 (P70), IL-13, IL-15, IL-17, KC, LIF, LIX, M-CSF, MIG, and VEGF. Remarkably, the release of CCL3, also called MIP-1α, was the strongest with around 50 ng/10^6^ cells; much higher than the previously established target of c-di-AMP, IFNβ was at around 4 ng/10^6^ cells. CCL4, CXCL2, and CXCL10 were also released to a larger extent than IFNβ. This experiment was reproduced independently for at least three more times, and additional controls were added such as untreated cells or cells treated with c-di-AMP alone to confirm the exclusive intracellular mode of action of c-di-AMP. Indeed c-di-AMP was only capable of significant induction in the presence of PS (Supplemental Figure 1). Moreover, BMDM were used to prepare total RNA and to perform RT-PCR to assess the cellular levels of induced mRNAs (Figure 1B). The levels of IFNβ and IL6 mRNAs increased dramatically compared to levels without c-di-AMP (more than 1,000-fold of abundance in PS-treated control cells). Based on RT-PCR observations, the mRNA levels of CCL3 in untreated cells were much higher than those of IL6 and IFNβ (Ct values in the range of 25 compared to 35 for IFNβ and IL6). Accordingly, the induction is strong even if it increased only 3- to 4-fold after 4 h of treatment. The same experiment was performed in the RAW264.7 macrophage-like mouse cell line with similar results (Figures 1C,D; Supplemental Figure 1). Overall the results were comparable between RAW264.7 cells and primary cells, indicating that this cell line is suitable for the investigation of c-di-AMP response. In an independent experiment, we compared the intracellular induction by c-di-AMP of IFNβ, IL6, CCL3, CCL4, COX2 (PTGS2), and TNF with the extracellular induction with LPS in RAW264.7 cells by RT-PCR (Supplemental Figure 2A). It turned out that c-di-AMP is a more potent inducer of IFNβ, IL6, and CCL4, while LPS induced CCL3 and TNF more potently. Unlike LPS, c-di-AMP could not induce COX2 in neither RAW264.7 (Supplemental Figure 2A) nor BMDM (data not shown).

### p38 MAPK Inhibition Reduces the Expression of c-di-AMP-Induced Cytokines

**Figure 2:**
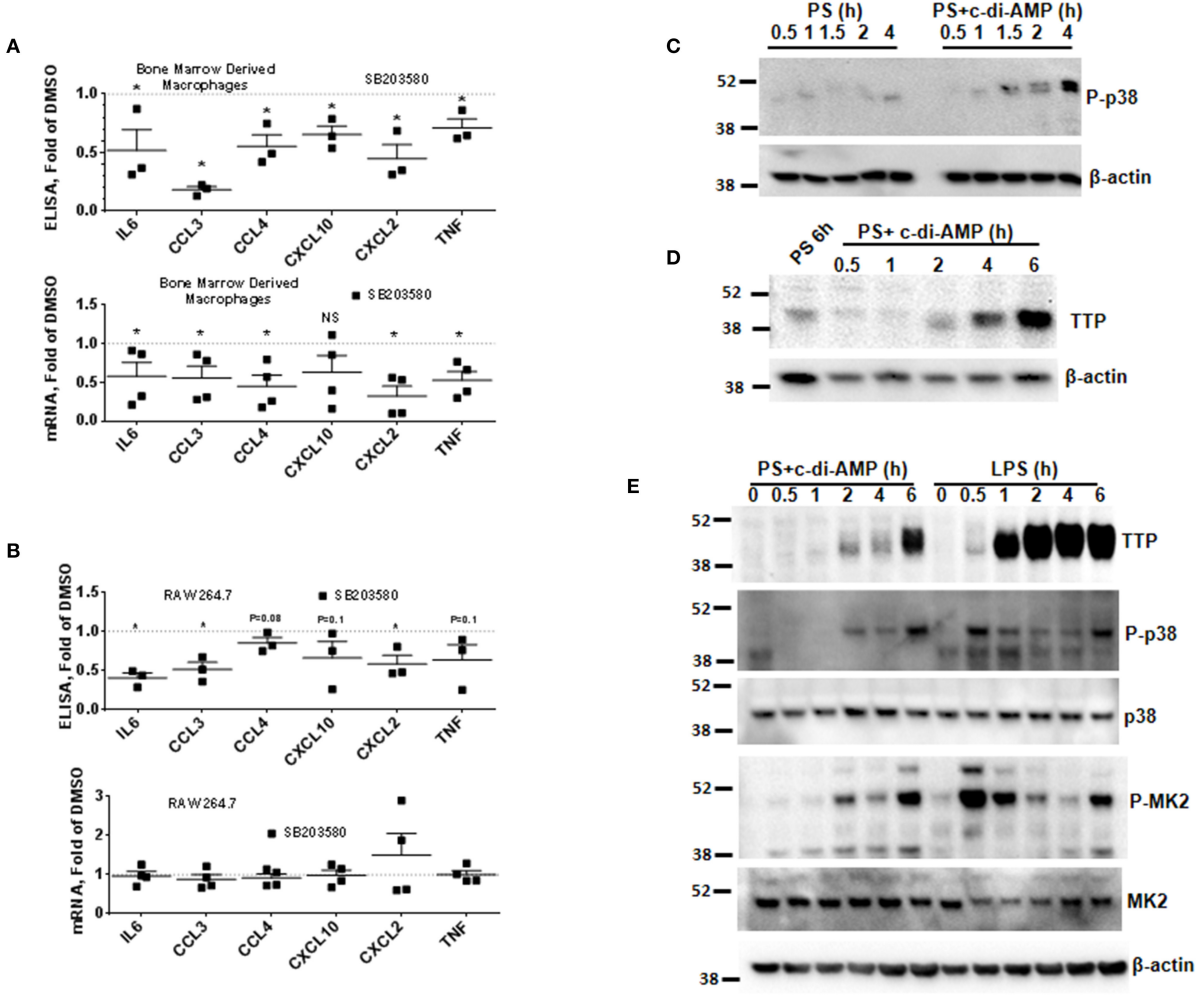
P38 inhibition leads to the reduction in the expression of ARE-containing mRNAs and induction of TTP. **(A)** Upper panel multiplex ELISA for the indicated cytokines. BMDM were treated with 5 μM SB203580 or DMSO during the treatment with c-di-AMP for 4 h. Cell medium was used for multiplex ELISA. Lower panel the BMDM were lysed, total RNA was prepared, and the mRNA levels of the indicated cytokines were determined. Individual values of at least three independent experiments are represented as fold of levels in DMSO control treated cells. Means ± SEMs are shown. *Asterisk* represents *P* <0.05 of paired *t*-tests. **(B)** Similar experiment like **(A)** but with RAW264.7 cells. **(C)** RAW264.7 cells were treated with PS alone or PS in the presence of c-di-AMP for the indicated time points. The cells were lysed in SDS loading buffer, and western blots were performed and developed with an antibody specific for phosphorylated p38. β-Actin was used as loading control. **(D)** Western blot similar to **(C)** with one PS control at 6 h and at time points as indicated. The blot was developed with anti-TTP antibody and β-actin. **(E)** RAW264.7 cells were treated for the indicated time points with PS+c-di-AMP or LPS. The cells were lysed in SDS loading buffer, and western blots were performed and developed with antibodies specific for TTP, phosphorylated p38 (P-p38), p38, phosphorylated MK2 (P-MK2), MK2, and β-actin.

The mRNAs of the c-di-AMP-induced cytokines IL6, CCL3, CCL4, CXCL2, and TNF contain AREs in their 3′UTR (Supplemental Figure 2B) (12). AREs are known to regulate cytokine expression at the post-transcriptional level by responding to p38 MAPK cellular signaling cascade. To investigate if p38 regulates the expression of the cytokines induced by c-di-AMP, BMDM were treated with the p38 inhibitor SB203580. The cytokine release of IL6, CCL3, CCL4, CXCL2, and TNF was significantly reduced by p38 inhibition (Figure 2A, upper panel). For instance, the amount of CCL3 levels dropped down to 70%. The corresponding mRNA levels were also significantly reduced (Figure 2A, lower panel). The same experiment was repeated with RAW 264.7 cells. A trend or significant reduction in the release was observed for most cytokines, while SB203580 inhibition had no significant effect on the mRNA levels (Figure 2B).

### c-di-AMP Induces TTP and Phosphorylation of p38 MAPK

**Figure 3:**
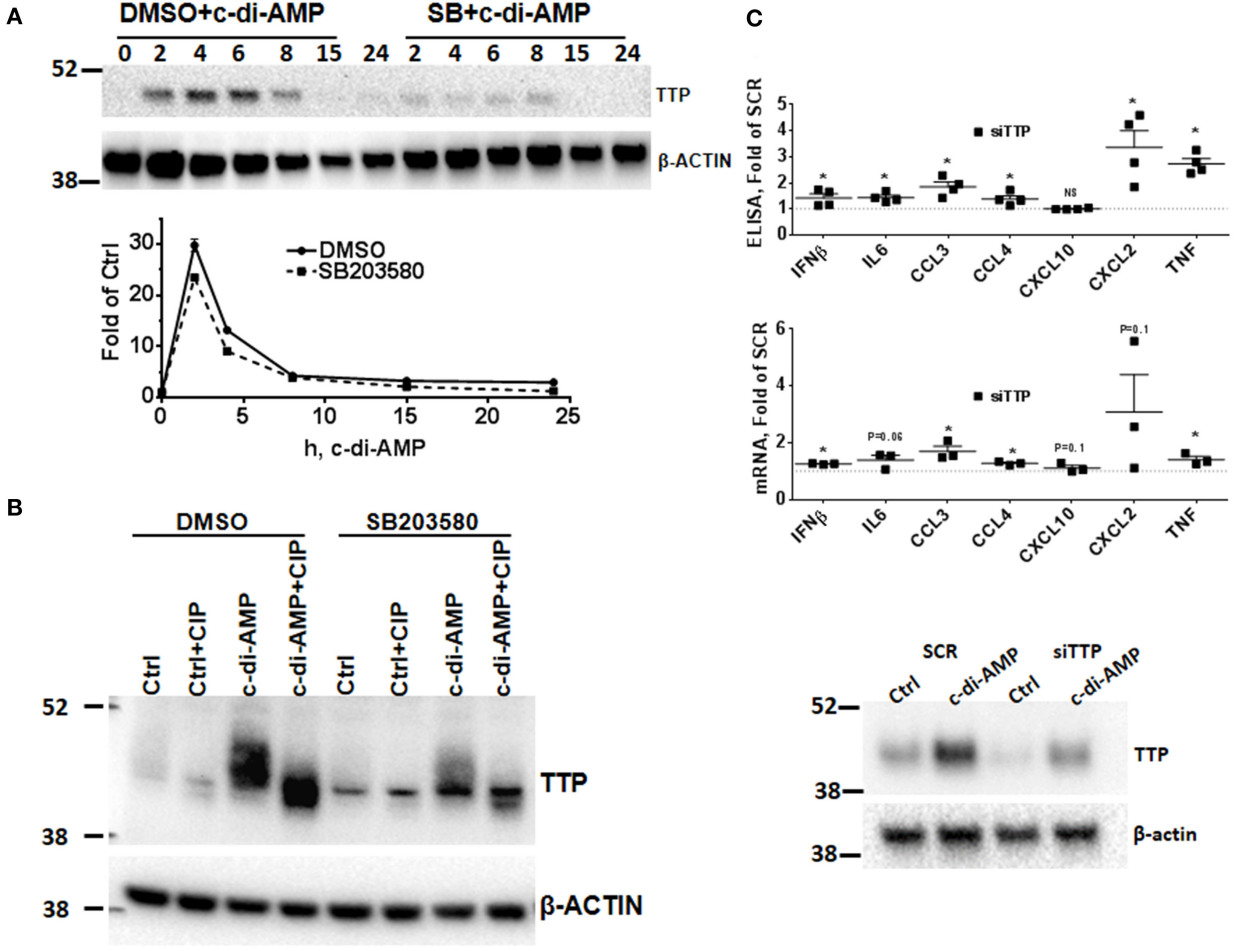
Effect of p38 inhibition on c-di-AMP induced-TTP and the consequence of its knockdown. **(A)** Upper panel RAW264.7 cells were treated with PS with c-di-AMP in the presence of DMSO or SB203580 for the indicated time points. Cells were then lysed in SDS loading buffer, and western blots were performed and developed with antibodies specific for TTP and β-actin. Lower panel same like in **(A)**, but total RNA was prepared and the levels of TTP mRNA were determined by RT-PCR. One experiment is shown; other independent experiments were performed with similar results. **(B)** RAW264.7 cells were treated with either PS alone (*Ctrl*) or PS and c-di-AMP for 5 h. The cells were lysed in CIP buffer supplemented with 0.5% NP40. Control (*Ctrl*) and lysates from c-di-AMP-treated cells were treated with CIP for 30 min, and long-run western blot was performed and developed with antibodies specific for TTP and β-actin. **(C)** RAW264.7 cells were transfected with either SCR control or siRNA pool that targets TTP prior to intracellular c-di-AMP treatment for 4 h. Upper panel supernatants were recovered and subjected to multiplex ELISA. Middle panel the cells were lysed, total RNA was prepared, and the mRNA levels of the indicated cytokines were determined. Individual values of at least three independent experiments are represented as fold of expression in SCR transfected cells. Means ± SEMs are shown. *Asterisk* represents *P* <0.05 of paired *t*-tests. Lower panel the cells were lysed, and western blot was performed to confirm the knockdown of TTP.

The p38 MAPK signaling pathway enhances the expression of inflammatory ARE-containing mRNAs by modulating the level and activity of the ARE-binding protein TTP (25, 27). To investigate if this pathway can be activated by intracellular c-di-AMP, we treated RAW 264.7 macrophage with PS alone or with PS and c-di-AMP for different time periods. The cells were lysed, and western blots were performed with antibodies that recognize the phosphorylated form of p38 (P-p38). Intracellular c-di-AMP was able to induce the phosphorylation of p38 starting at 60 min but was stronger at 4 h (Figure 2C). The induction of TTP was visible after 2 h but was stronger at 6 h (Figure 2D). Next, the ability of c-di-AMP to induce TTP and trigger the phosphorylation of p38 and MK2 was compared with LPS by western blot using total and phosphospecific antibodies (Figure 2E). It turned out that LPS is a much stronger inducer of TTP, and the kinetics of phosphorylation of p38 and MK2 are different between LPS and c-di-AMP. With LPS, the phosphorylation of both p38 and MK2 peaks at 0.5 h, while with c-di-AMP it starts at 0.5 h but is stronger at 6 h post-treatment. Previous reports demonstrated that the p38/MK2 signaling cascade that is triggered by LPS inhibits the activity but enhances the expression of TTP by protein stabilization due to direct phosphorylation (23–25, 33). To investigate if a similar phenomenon is taking place when TTP is induced by c-di-AMP, we treated cells with intracellular c-di-AMP for 2 h and up to 24 h in the presence or absence of the p38 inhibitor SB203580. Similar to the LPS situation, TTP levels were reduced but probably also activated due to reduced phosphorylation (Figure 3A, upper panel). Similarly, the levels of induced TTP mRNA were reduced in p38 MAPK inhibition background (Figure 3A, lower panel). To investigate if c-di-AMP-induced TTP is phosphorylated, protein lysates were treated with CIP. CIP treatment led to a downshift of the TTP bands on a long-run western blot, indicating phosphorylation (Figure 3B). Treatment with SB203580 led to a reduction in the level of the slow-migrating, highly phosphorylated bands, implying phosphorylation through the p38 pathway that is activated by c-di-AMP (Figure 3B).

### TTP Knockdown Increases c-di-AMP-Dependent Cytokine Production

Since TTP is induced by c-di-AMP, the effect of TTP knockdown KD on induced cytokines was assessed by multiplex Luminex bead assay. The KD had a strong impact on CCL3 production which increased up to 2-fold. CCL4, IFNβ, CXCL2, and TNF levels were also up-regulated, while CXCL10 levels were not affected by the KD (Figure 3C, upper panel). Steady-state mRNA levels were also evaluated, and most were increased when TTP levels were knocked down (Figure 3C, middle panel). The knockdown of TTP was assessed by western blot (Figure 3C, lower panel). This result is in agreement with the established effect of TTP knockout on the production of cytokines after LPS induction (27, 34).

### Time Course of c-di-AMP-Induced Cytokine mRNA in p38 Inhibition and TTP KD Background

**Figure 4:**
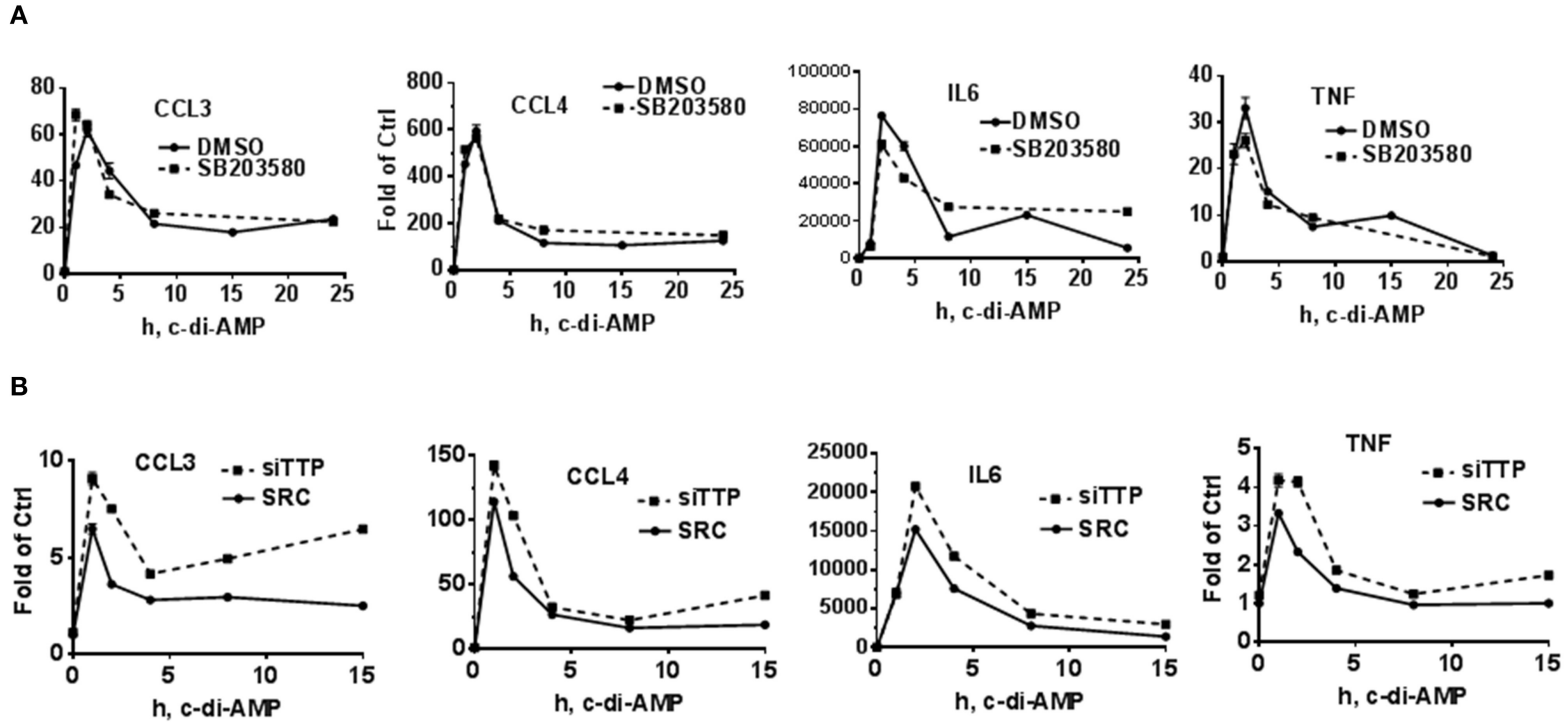
Time course of c-di-AMP-dependent induction of cytokines in a p38 inhibition and TTP knockdown background. A total of 5 × 10^5^ RAW264.7 cells were seeded in six-well plates and treated with intracellular c-di-AMP for the indicated time points. Total RNA was prepared, and RT-PCR was performed for the indicated cytokines. **(A)** The cells were treated with DMSO vehicle or 5 μM SB203580. **(B)** The cells were either transfected with SCR control or siRNA pool that knocks down TTP levels prior to the c-di-AMP treatment for the indicated time points. One experiment is presented. Two additional experiments were performed with comparable results.

To further investigate a possible effect of p38 inhibition or TTP KD on the levels of the mRNA of c-di-AMP-induced cytokines, time course experiments were performed in RAW264.7 cells. For p38 inhibition, the cells were treated with either DMSO as vehicle control or with 5 μM of SB203580. The addition of the inhibitor started together with c-di-AMP and PS. The mRNA levels of CCL3, CCL4, IL6, and TNF were measured. For all four cytokine mRNAs, the levels of induction peaked at 1 h post-treatment and returned to near-pre-treatment levels after 8 h, indicating a transient nature of the induction. The inhibition of p38 did not have major or reproducible effects on the mRNA steady-state levels of the cytokines (Figures 2B, 4A). For the effect of TTP KD, the cells were either transfected with siRNA control or siTTP in 10-cm plates before reseeding into six-well plates to ensure homogenous levels of transfection between the time points. In the SCR transfection control, the time course curves were in general similar to the p38 inhibition for all cytokines. TTP KD resulted in higher steady-state levels of the investigated cytokine mRNAs (Figure 4B).

### c-di-AMP-Induced Cytokine mRNA Stability After p38 Inhibition and TTP Knockdown

**Figure 5:**
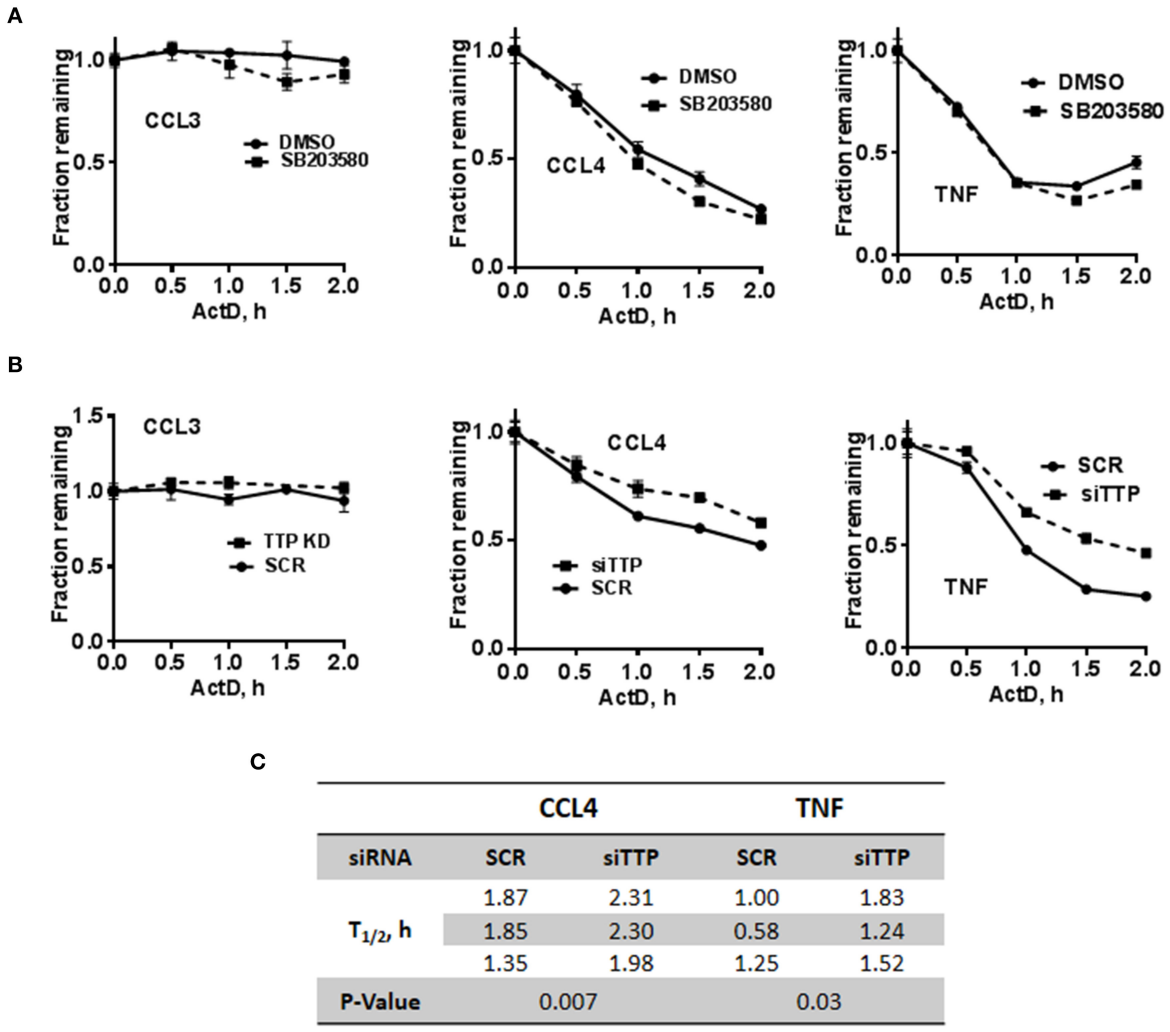
Stability of c-di-AMP-induced cytokine mRNAs after TTP KD or p38 inhibition. **(A)** A total of 5 × 10^5^ RAW264.7 cells were seeded in six-well plates and treated with intracellular c-di-AMP for 4 h together with DMSO vehicle or 5 μM SB203580, then with 10 μg/ml actinomycin-D for the indicated time points. Total RNA was prepared, RT-PCR was performed for the indicated cytokines, and the decay curves were plotted using graph prism. Estimated half-life is the intersection point between the decay curve and the 0.5 fraction remaining. **(B)** Same as A with cells that were transfected with either SCR control or siRNA that targets TTP prior to the c-di-AMP treatment. **(C)** Half-lives of CCL4 and TNF mRNAs were determined in three independent experiments, and statistical significance was determined by paired *t*-test.

The stability of c-di-AMP-induced CCL3, CCL4, and TNF was assessed with or without p38 inhibition by performing Actinomycin-D chase experiments (Figure 5A). The SB203580 inhibitor or vehicle was added together with the c-di-AMP and PS for 4 h before adding Actinomycin-D, as described in the “Materials and Methods” section. Treatment with SB203580 had in some experiments a slight but not reproducible effect on the stability of CCL3, CCL4, and TNF mRNAs, and statistical significance could not be reached even after six attempts (Figure 5A).

TTP KD did not have a major effect on the stability of CCL3 since it is already stable even without the KD. The half-lives of CCL4 and TNF mRNAs were estimated in three independent experiments and were significantly higher when TTP was knocked down (Figures 5B,C).

### c-di-AMP Up-Regulates the Expression of an ARE-Containing Nano-Luciferase Reporter

**Figure 6:**
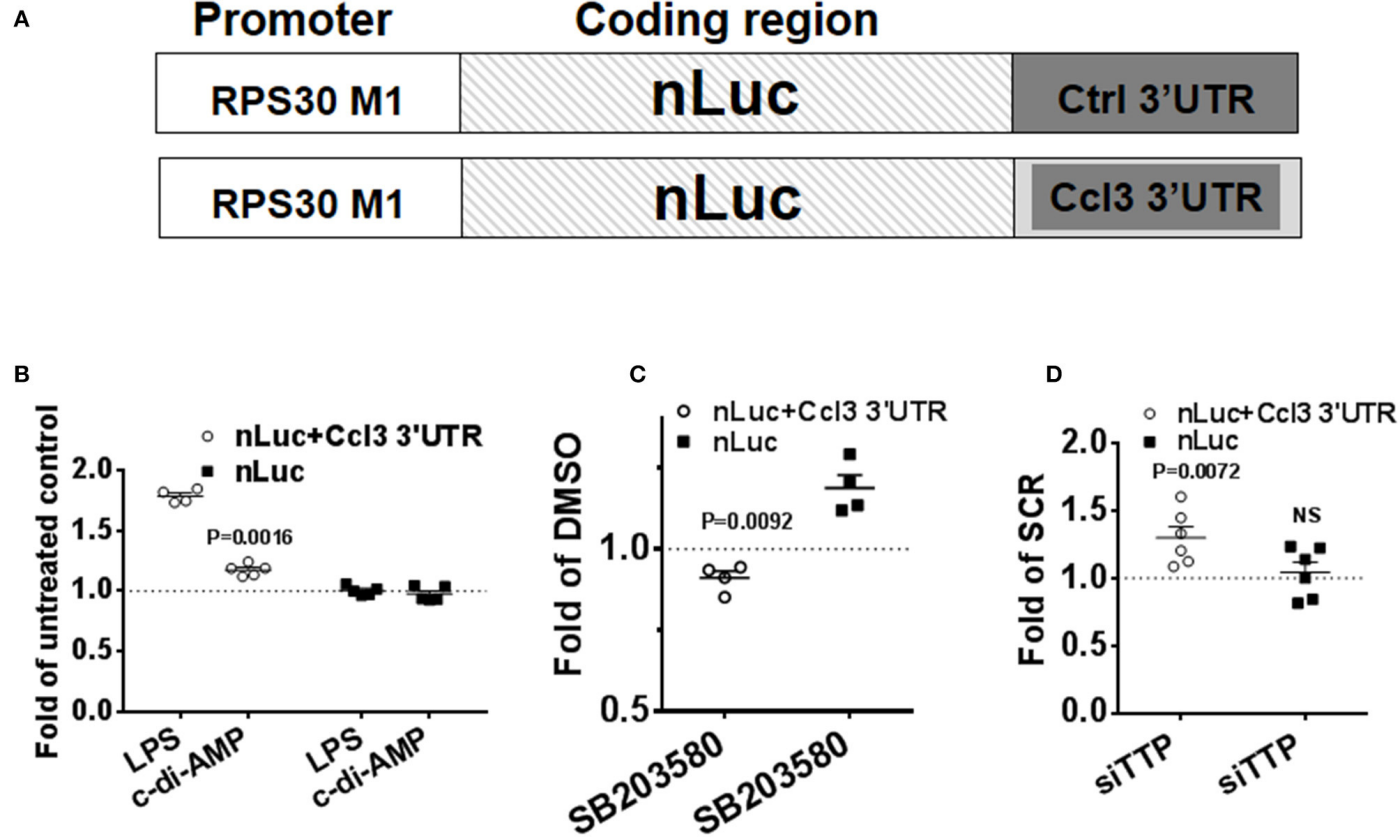
Specific up-regulation of the expression of a reporter that contains an ARE by c-di-AMP. A total of 5 × 10^5^ RAW264.7 cells were co-transfected with firefly transfection control plasmid (*FF*) and either with a reporter that contains the 3′UTR of Ccl3 as an insertion in its 3′UTR (*nLuc*+*Ccl3 3*′*UTR*) or a control nano-Luciferase reporter (*nLuc*). **(A)** Schematic representation of the transfection nLuc reporters. **(B)** The cells were either left untreated or were treated with LPS or treated with PS alone or PS with c-di-AMP for 8 h before lysis and nano-Luciferase and FF activity measurements. The results for LPS treatment are shown as fold of untreated cells, while for c-di-AMP, results are shown as fold of PS-treated cells. **(C)** nLuc+Ccl3 3′UTR or nLuc-transfected cells were treated with intracellular c-di-AMP for 8 h in the presence of DMSO or 5 μM SB203580. The effect of SB203580 on the expression of the reporter is presented as fold of the reporter expression in DMSO-treated cells. **(D)** RAW264.7 cells were cotransfected with nLuc+Ccl3 3′UTR or nLuc with either scramble dsRNA (*SCR*) or siRNA that targets TTP. The cells were treated with intracellular c-di-AMP for 8 h before measurement of reporter expression. The effect of TTP knockdown on the expression of the reporter is presented as fold of reporter expression in SCR-transfected cells. Individual values of at least four independent experiments are represented. Means ± SEMs are shown. *P*-values of paired *t*-tests of the relevant results are shown.

To confirm the post-transcriptional mode of action of c-di-AMP, nLuc reporter assays were performed. The 3′UTR of *CCL3* mRNA was cloned into the nLuc expression vector that is under the control of a non-inducible RPS30 promoter that is suitable for the investigation of post-transcriptional activities (Figure 6A) (27, 35). The cells were transfected in 10-cm plates and re-seeded into six-well plates to ensure homogenous levels of transfection. FF reporter was used for transfection normalization, and the results were normalized to control. LPS treatment for 8 h was used as positive control, and the reporter that contains the 3′UTR of *CCL3* responded by ~50% increase in the reporter activity (Figure 6B). The non-ARE reporter (nLuc) did not respond to LPS (Figure 6B). Then, we compared the level of expression between PS-treated cells and cells that were treated with PS and c-di-AMP. A statistically significant ~25% c-di-AMP-dependent up-regulation of the reporter activity was observed only in the presence of the 3′UTR of *CCL3* (Figure 6B). The non-ARE reporter did not respond to c-di-AMP (Figure 6B). These results clearly indicate that c-di-AMP induces the expression of ARE-containing cytokine mRNA at the post-transcriptional level. The inhibition of p38 MAPK with SB203580 in c-di-AMP-treated cells led to a reproducible ~10% reduction in the expression of nLuc+Ccl3 3′UTR compared to cells treated with vehicle (Figure 6C). This apparent slight reduction might be understated since treatment of the non-ARE reporter (nLuc)-transfected cells with SB203580 led to an unexpected reproducible up-regulation of expression (Figure 6C). The knockdown of TTP led to a specific and significant up-regulation in the expression of the ARE-containing reporter after intracellular c-di-AMP delivery (Figure 6D).

## Discussion

Host pattern recognition receptors bind conserved molecular structures found in infecting bacteria and microbes. These structures are called pathogen-associated molecular patterns (PAMPs), and one of the most investigated PAMPs is the bacterial LPS which induces strong innate immunity responses (36, 37).

The data presented here show that c-di-AMP of intracellular pathogenic bacteria, which is known to act as an intracellular PAMP, can induce a wider response in host macrophages than previously reported (2, 10). It is a potent inducer of a number of cytokines like IL6, CXCL2, CCL3, and CCL4 in addition to IFNβ. In fact, compared to extracellular LPS, c-di-AMP appears to be a stronger inducer of IFNβ, CCL4, and IL6, while LPS induces CCL3 and TNF to higher levels. Interestingly, c-di-AMP fails to induce the COX-2 (PTGS2) inflammatory gene, while LPS induces it potently (Supplemental Figure 2). This comparison with LPS unveils the strong potency of c-di-AMP as an inducer of innate immunity. The two PAMPs, LPS that acts extracellularly and c-di-AMP that acts intracellularly, induce signaling cascades with common but also different outcomes in terms of level and identity of induced inflammatory mediators. Here we report that the p38 MAPK cellular pathway, which is a master regulator of the post-transcriptional inflammatory response, is activated by both LPS and c-di-AMP (38, 39).

A clue for a post-transcriptional stimulation of ARE-containing mRNA cytokine expression by c-di-AMP came through the observation and comparison of the steady-state levels of IFNβ and IL6 with those of CCL3 or CCL4 mRNAs. IFNβ and IL6 mRNA levels are very low in untreated resting macrophages, typically around six orders of magnitude lower than the housekeeping β-actin gene according to the real-time PCR estimates performed in this study. The levels of steady-state CCL3 or CCL4 mRNA are comparatively much higher in resting cells, around four orders of magnitude higher than IFNβ and IL6 and just 5,060 times lower than β-actin mRNA. Even after stimulation, the levels of IL6 and IFNβ mRNA remain lower than the levels of CCL3 or CCL4 in unstimulated cells. Since unstimulated cells should not express CCL3 or CCL4, the high level of their mRNAs in resting cells must be repressed at the level of translation, and this has been previously reported (40). After a 4-h induction period with c-di-AMP, 5–15 ng/10^6^ cells of IFNβ and IL6 are released compared to 120 ng CCL3. At the mRNA level, IFNβ and IL6 increase more than 4,000-fold, while CCL3 is induced in the range of 10-fold. This observation suggested that the translation of CCL3 and CCL4 mRNAs must be released in response to c-di-AMP since a modest relative induction of its mRNA leads to a very strong release of the cytokine.

Indeed c-di-AMP turned out to be capable of activating the p38 MAPK, which includes the phosphorylation of p38 and induction and phosphorylation of TTP. Overall this activation is similar to that of LPS (41), albeit with at least one significant difference. The phosphorylation of p38 by LPS is transient, peaks at 30 min, and returns to near-background levels after only 1 h (42), whereas the p38 phosphorylation by c-di-AMP is slower and sustained; it starts early after treatment and continues to be strongly phosphorylated even after 4 h. This is in agreement with the effect of c-di-AMP on the expression of ARE-containing nLuc reporter which was more significant at 8 h post-treatment compared to earlier time points. Elements upstream of p38 in c-di-AMP-triggered signaling module are still unknown. However, intracellular receptors of other PAMPs such as RIG-I or NOD1 are capable of activating p38 (28, 30–32). Also, recent reports indicate that the cyclic dinucleotides from pathogens can directly bind to the oxidoreductase RECON and modulate the inflammatory response (43). Importantly, c-di-AMP triggers STING-cGAS signaling, resulting in the activation of interferon signaling (10, 44). Since interferons can activate p38 and the phosphorylation of p38 by c-di-AMP is delayed, an indirect activation may be possible (45, 46).

The inhibition of p38 or the knockdown of TTP had moderate effects on the steady-state levels and stabilities of the cytokine mRNAs tested but a pronounced effect on the release of the cytokines, again suggesting an effect on protein translation. A definite evidence for a post-transcriptional effect came from the reporter assay that contains the 3′UTR of CCL3 that specifically responded to c-di-AMP. We found a reproducible c-di-AMP-dependent 25% up-regulation of the ARE reporter, which is likely an underestimation since it had to be compared against cells treated with permeabilization solution which itself might cause stress responses in the cell, including a background activation of p38.

C-di-AMP is produced by intracellular bacterial pathogens like *Listeria monocytogenes* which can cause severe pathological conditions including sepsis (47). Therefore, a better understanding of the scope of the inflammatory response that is stimulated by c-di-AMP can contribute to better treatment.

## Data Availability Statement

All datasets generated for this study are included in the article/Supplementary Material.

## Ethics Statement

The animal study was reviewed and approved by the Animal Care and Use Committee, Office of Research Affairs, King Faisal Specialist Hospital and Research Center.

## Author Contributions

KK advised and conceived the original idea. EH designed the project and experiments and wrote the manuscript. LM and AA carried out and analyzed most of the experiments. SK, WM, and SA carried out and analyzed experiments. All authors reviewed and corrected the manuscript.

### Conflict of Interest

The authors declare that the research was conducted in the absence of any commercial or financial relationships that could be construed as a potential conflict of interest.
