# Supplementary figures and images for: Post-Transcriptional Inflammatory Response to Intracellular Bacterial c-di-AMP
Source: Front Immunol. 2020 Jan 17;10:3050. doi: 10.3389/fimmu.2019.03050 (PMC6979040; doi:10.3389/fimmu.2019.03050)

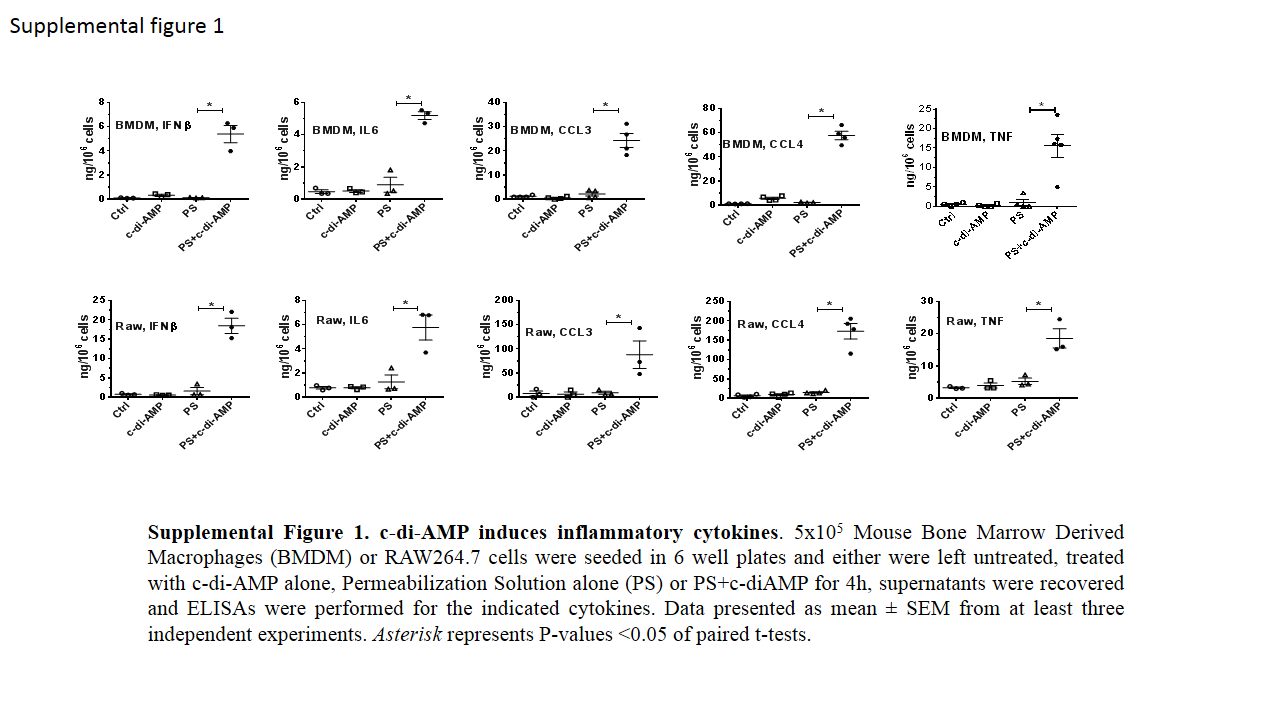

Supplement: Supplementary file 1 [file Image_1.tif]

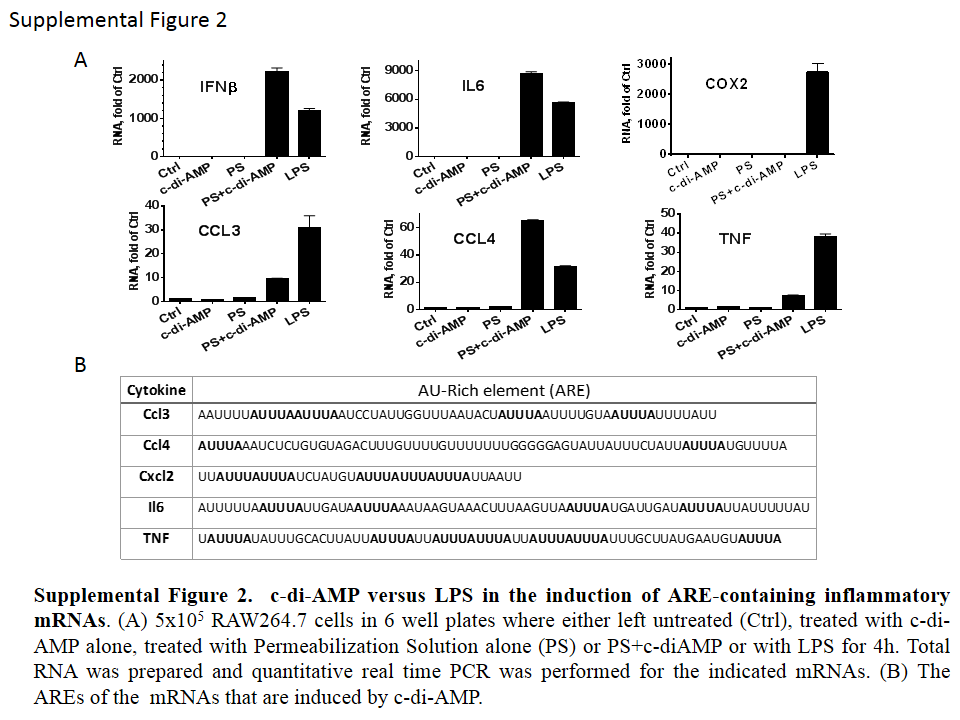

Supplement: Supplementary file 2 [file Image_2.TIF]
